# Comparison of cone beam computed tomography and plane radiographs of radial fractures as a basis for radiographical measurements

**DOI:** 10.1186/s12880-023-01093-4

**Published:** 2023-09-14

**Authors:** Kristian Bry, Mika Kortesniemi, Mika Koivikko, Liisa Kerttula

**Affiliations:** 1 Bridge Hospital, HUS, Haartmaninkatu 4, 00029 Helsinki, Finland; 2 Department of Radiology, Helsinki Medical Imaging Center, Helsinki University Hospital and University of Helsinki, 00029 Helsinki, Finland; 3 Department of Radiology, Medical Imaging Center, University of Helsinki and Helsinki University Hospital, P.O.Box 263, HUS, 00029 Helsinki, Finland

**Keywords:** Cone-beam computed tomography, Radiography, Radius fractures

## Abstract

**Background:**

The purpose of this study was to determine whether radiological measurements of radial fracture position made in cone beam computed tomography (CBCT) projection images are comparable to those made on traditional radiographs and could potentially substitute them.

**Methods:**

Sixteen patients with fractures of the distal radius referred for radiographs were recruited for an additional CBCT scan which was performed immediately afterwards. Projection images and volumetric data were saved from the CBCT scans. Measurements of ulnar variance, radial inclination and volar tilt were made from all three sets of images.

**Results:**

Agreement of projection image based measurements with radiographs was nearly as good as as the agreement of cross sectional image measurements with radiographs. The average difference between the results for projection images and radiographs were -1.2 mm (SD 1.9 mm), for radial inclination 0.7° (SD 2.9°) and for volar tilt 1.9° (SD 5.6°).

**Conclusion:**

Differences between radiological measurements between the modalities studied are small and projection images could be used for the assessment of distal radial fractures.

**Supplementary Information:**

The online version contains supplementary material available at 10.1186/s12880-023-01093-4.

## Background

Traditionally diagnosis of wrist fractures has been based on radiographs, and along with the clinical information, measurements made on radiographic images play a role in clinical decision making. Radiographic measurements such as ulnar variance, radial inclination and volar tilt are used clinically to determine treatment (conservative vs. operative) of distal radial fractures [1–3].

However, clinically relevant injuries are often missed on conventional radiography, so computed tomography (CT) is often necessary [4–6]. Due to the nature of three-dimensional (3D) image data, CT demonstrates occult fractures with greater sensitivity and provides more information about fracture morphology and joint surface affliction than conventional radiographs [7]. In addition to multi-slice CT, cone beam computed tomography (CBCT) is used for diagnosis of peripheral fractures [8–10]. Following the basic principle in CT imaging, CBCT is a cross-sectional imaging technique based on numerous planar projection images (PI) taken in an arc around the field of view using a cone shaped x-ray beam. These raw-data images are used to create volumetric data [11]. Typically, CBCT scans are interpreted based on the 3D images reconstructed from these projection images. However, the acquired raw-data projection images themselves could also be used for diagnostics and to guide clinical decision making.

Because these CBCT projection images are two dimensional (like radiographs), they could potentially serve as a substitute for radiographs for certain clinical purposes, for example as a template for radiographic measurements, which, to our knowledge, has not previously been investigated. With increasing work-load for radiologists and clinicians alike, fast and pragmatic image interpretation is becoming critical and superfluous imaging studies should be avoided. The purpose of this study was to ascertain to what degree measurements made from CBCT projection images are comparable to measurements made on ordinary radiographs or CBCT cross sectional images.

## Methods

The study was conducted at Töölö Hospital, a level one trauma center in Helsinki, Finland. The study was approved by the Surgical Ethical Review Board of the Hospital District of Helsinki and Uudenmaa (approval reference number 118/13/03/02/2015) and the study was conducted according to the Helsinki Declaration. Informed consent was obtained from all individual participants included in the study. Sixteen outpatients with distal radial fractures who were referred to radiographs in 2015–2016 were recruited for an additional CBCT scan.

CBCT scans were performed immediately after the wrist radiograph. The CBCT scans were performed with the wrist in neutral position. CBCT projection images and cross sectional images were obtained from the same CBCT scan.

### Information about scanners

The CBCT scanner used in this study was the Carestream OnSight 3D Extremity System (Carestream Health Inc., Rochester, NY, USA). The CBCT scanner includes three fixed-anode x-ray tubes with 0.5 mm focus spot size. Three separate x-ray tubes are used in the CBCT scanner to avoid the cone-beam artefacts in both ends of the vertical scan range. The operational range of the x-ray tubes are 50–90 kV tube voltages and 2–10 mA tube currents. The tube voltage of 90 kV and 5 mA tube current was used in the CBCT scans in this study. The x-ray beam filtration was 2.5 mm-Al with 0.1 mm Cu added filtration. The estimated radiation dose is 0.01 mSv/mGycm^2^. The raw data projections were acquired with an amorphous silicon digital flat panel CsI(Tl) detector (Varian PaxScan 2530DX) which has 139 µm element size and 2 × 2 binning used in the scans. The field-of-view used in the study was 219 mm wide and 216 mm in height. The scan time of around 25 s included an effective x-ray exposure time of 6 s for a 215 degrees rotation angle covered in the raw data projection image acquisition. The image matrix of 884 × 1076 pixels were used in the CBCT projection images. The cross-sectional image slice thickness was 0.2604 mm with image matrix size of 884 × 884 pixels in the CBCT 3D image data.

### Image analysis

Two radiologists, a fifth year radiology resident and an experienced musculoskeletal radiologist with fifteen years of subspecialty experience, independently analyzed the images with an interval of at least one to two weeks between the different modalities. Images were reviewed in randomized order. Measurement from projection images and radiographs were performed on an Impax workstation (Impax 6.6, Agfa-Gaevert, Mortsel, Belgium). Reconstructions and measurements from volume images were made using Vitrea (Vitrea 6.9.87.1, Vital Images Inc. Minnetonka, MN) using averaged or maximum intensity projection (MIP) reformatted images when appropriate to identify the necessary anatomical landmarks.

When measuring from the CBCT projection images, each observer chose the images which were considered to best correspond to posterioanterior and lateral radiographs. The radiographical parameters analyzed were ulnar variance (UV), radial inclination (RI), and volar tilt (VT). Ulnar variance was defined as the long axis distance between the most distal extent of the ulnar head and the sigmoid notch (positive if the ulna was longer than the ulnar corner of the radius). Radial inclination was defined as the angle between a line perpendicular to the long axis of the radius and a line between the ulnar aspect of the articular corner of the distal radius and the tip of the radial styloid process. Volar tilt was defined as the angle between the dorsal and volar corners of the distal radial articular surface and a line perpendicular to the long axis of the radius on a lateral view (the value was negative if the radial articular surface was dorsally tilted) [1, 6].

**Fig. 1:**
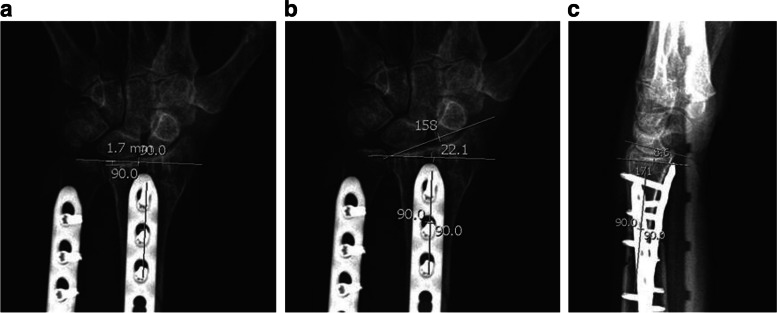
**a-c** Images showing projection images with measurements of (**a**) ulnar variance, (**b**) radial inclination, and (**c**) volar tilt

Example images of each measurement projections images for the same patient are shown in Fig. 1.

In addition to these radiological measurements, the radiologists independently measured the off-centricity of the images: the distance from the radiocarpal joint to the upper and left edges of the projection images. They also measured the angle between longitudinal axis of the radius and the z-axis of the scanner from both PA- and lateral PI projections. They also recorded which projection image each measurement was made from. After collection of measurements, obvious errors in the data (e.g. incorrect sign, typographic errors) were corrected by consensus.

### Statistical analysis

Statistical analysis was performed using Microsoft Excel (Microsoft Corporation, Redmond, WA) and SPSS Statistics (IBM Corporation, Armonk, NY). The average values and standard deviations for each measurement were calculated, as well as the average differences between the three modalities for each measurement. In addition, the average inter-observer variation was calculated for each measurement and modality. The measurements made in each modality were plotted against the corresponding measurements in each of the other two modalities and a linear regression line was calculated. The agreement of the results for each measurement for projection images and cross sectional images were compared to the results for radiographs (the clinical standard) using Bland–Altman plots.

## Results

The imaging studies were technically successful. Three (18.8%) of the CBCT studies had slight motion artefacts visible in the cross sectional images, but these artefacts were insignificant.

**Fig. 2:**
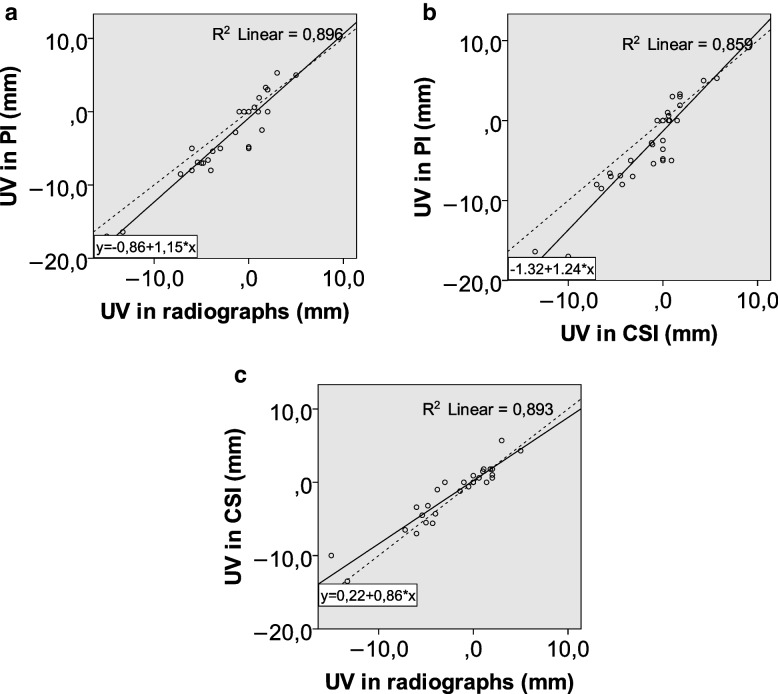
**a**-**c** Plots showing the results for ulnar variance for both observers in radiographs, projection images, and cross sectional images plotted against each other, with lines of best fit and r.^2^ values. The dashed line represents an x = y line.(UV ulnar variance, PI projection images, CSI cross-sectional images)

**Fig. 3:**
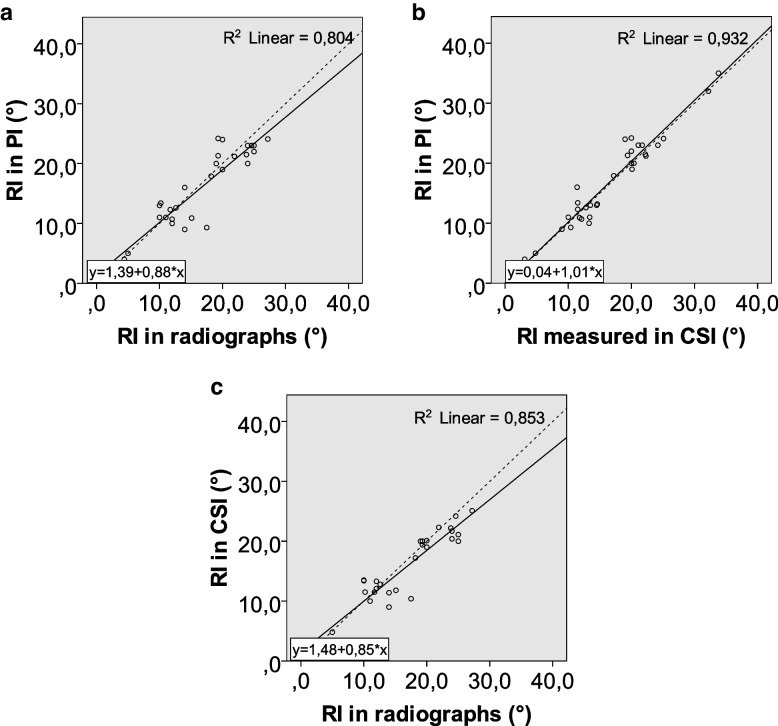
**a**-**c** Plots showing the results for radial inclination for both observers in radiographs, projection images, and cross sectional images plotted against each other, with lines of best fit and r.^2^ values. The dashed line represents an x = y line. (RI radial inclination, PI projection images, CSI cross-sectional images)

**Fig. 4:**
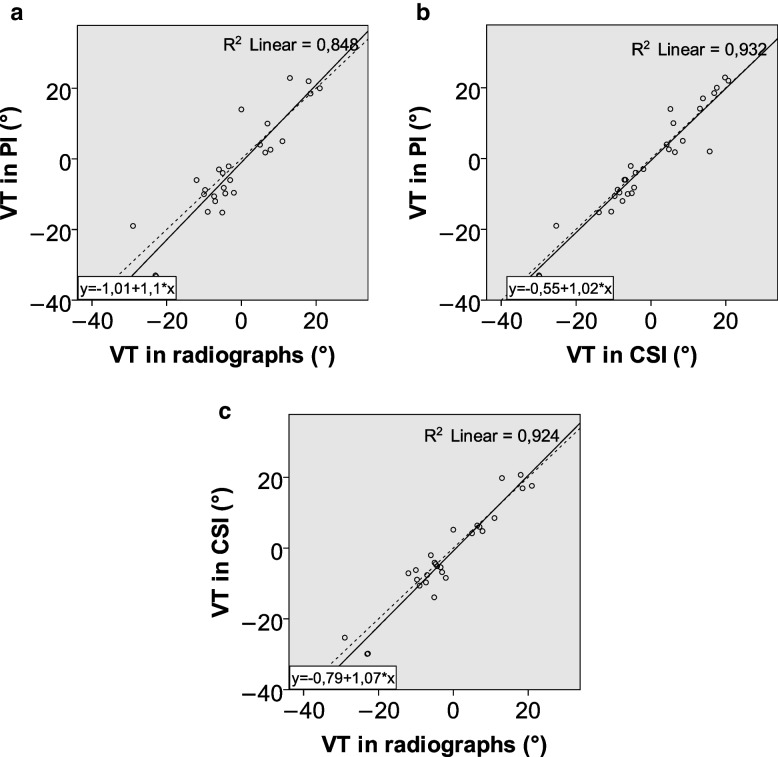
**a-c** Plots showing the results for volar tilt for both observers in radiographs, projection images, and cross sectional images plotted against each other, with lines of best fit and r.^2^ values. The dashed line represents an x = y line. (VT volar tilt, PI projection images, CSI cross-sectional images)

Clinical characteristics of patients are summarized in Table 1. The average results and standard deviations for each of the imaging parameters are reported in Table 2. Inter-observer variation is reported in Table 3. Average differences between modalities for each parameter are reported in Table 4. Plots of the results for each measurement and modality with linear correlation lines are shown in Figs. 2, 3 and 4.

**Table 1:** Clinical characteristics of patients

Operative fixation	6 (37.5%)
volar plate	4
dorsal plate	1
diaphyseal plate	2
Cast	11 (68.7%)
Average age	53.9 (SD 16.1)
Women	93.8% (n = 15)

**Table 2:** Average measurements and standard deviations (in parentheses) for each parameter by modality (PI projection images, UV ulnar variance, RI radial inclination, VT volar tilt)

	Average UV [mm]	Average RI [°]	Average VT [°]
PI	3.1 (5.4)	17.1 (7.3)	-2.8 (15.7)
Radiographs	2.2 (4.7)	16.8 (6.4)	-3.1 (13.4)
Cross-sectional images	1.4 (4.0)	16.8 (6.9)	-1.8 (14.9)

**Table 3:** Inter-observer variation (standard deviation in parentheses)

	Average inter-observer difference (projection images)	Average inter-observer difference (cross-sectional images)	Average inter-observer difference (radiographs)
Ulnar variance [mm]	-0.5 (1.1)	-0.3 (1.3)	-0.3 (1.0)
Radial inclination [°]	0.2 (2.4)	0.5 (2.2)	0.3 (1.5)
Volar tilt [°]	1.9 (4.0)	1.9 (2.1)	0.6 (3.7)

**Table 4:** Average differences between modalities for each parameter and standard deviations in parentheses. (PI projection images, CSI cross sectional images, SD standard deviation)

	Average difference between radiograph and PI (SD)	Average difference between CSI and PI (SD)	Average difference between radiograph and CSI (SD)
Ulnar variance [mm]	-1.2 (1.9)	-1.7 (2.3)	0.5 (1.5)
Radial inclination [°]	0.7 (2.9)	0.3 (1.9)	1.0 (2.4)
Volar tilt [°]	1.9 (5.6)	0.7 (4.1)	1.2 (4.0)

**Fig. 5:**
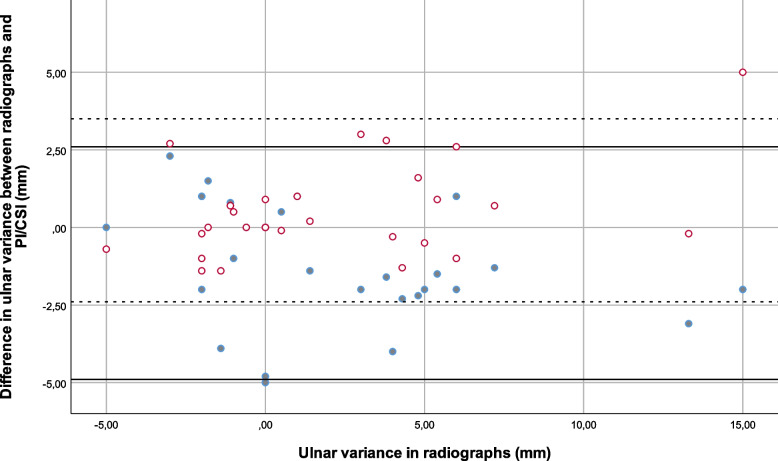
Bland–Altman plot comparing the differences between results for ulnar variance in projection images versus radiographs (solid dots) and cross-sectional images versus radiographs (hollow dots), with lines at at ± 1.96 SD (95% limits of agreement) (solid lines are the difference between PI’s and radiographs and dashed lines the difference between CSI’s ja radiographs). (UV ulnar variance, rad radiographs, CSI cross sectional images, PI projection images)

**Fig. 6:**
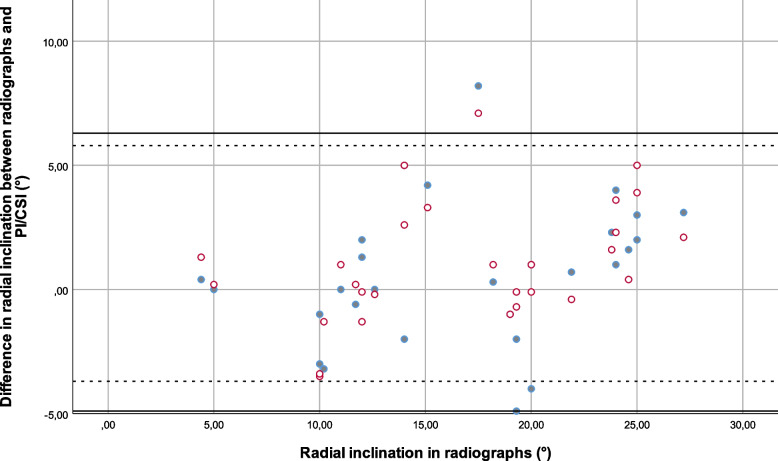
Bland–Altman plot comparing the differences between results for radial inclination in projection images versus radiographs (solid dots) and cross-sectional images versus radiographs (hollow dots), with lines at at ± 1.96 SD (95% limits of agreement) (solid lines are the difference between PI’s and radiographs and dashed lines the difference between CSI’s ja radiographs). (RI radial inclination, rad radiographs, CSI cross sectional images, PI projection images)

**Fig. 7:**
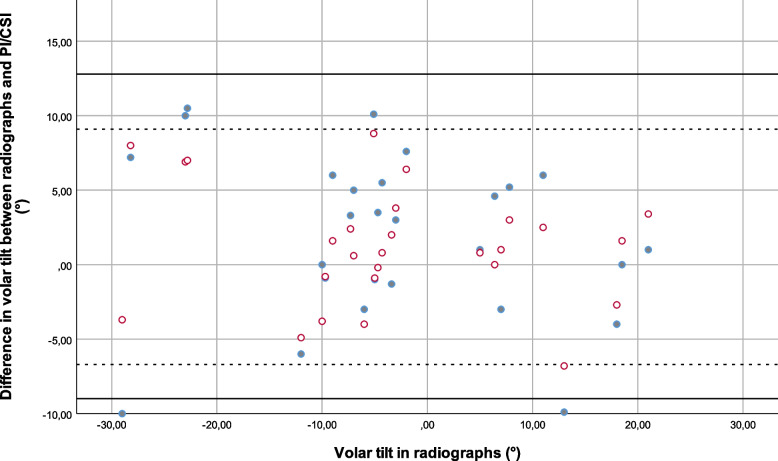
Bland–Altman plot comparing the differences between results for volar tilt in projection images versus radiographs (solid dots) and cross-sectional images versus radiographs (hollow dots), with lines at at ± 1.96 SD (95% limits of agreement) (solid lines are the difference between PI’s and radiographs and dashed lines the difference between CSI’s ja radiographs). (VT volar tilt, rad radiographs, CSI cross sectional images, PI projection images)

The Bland–Altman plots showing the agreement of the results for projection images and cross sectional images compared to radiographs are shown in Figs. 5, 6 and 7.

The mean difference between projection images and radiographs (the clinical standard) for ulnar variance was -1.2 mm and the 95% confidence interval according to Bland–Altman was -4.9 – 2.6 mm; for radial inclination the mean difference was 0.7° and the Bland–Altman 95% confidence interval was -4.9 – 6.3°; and for volar tilt the mean difference was 1.9° and the Bland–Altman 95% confidence interval was -9.0 – 12.8°.

The mean difference between cross-sectional images and radiographs (the clinical standard) for ulnar variance was 0.5 mm and the Bland–Altman 95% confidence interval was -2.4 – 3.5 mm; for radial inclination the mean difference was 1.0° and the Bland–Altman 95% confidence interval was -3.7 – 5.8°; and for volar tilt the mean difference was 1.2° and the Bland–Altman 95% confidence interval was -6.7 – 9.1°.

The 95% confidence intervals were thus somewhat broader for projection images compared to radiographs than for cross-sectional images compared to radiographs.

The absolute value of the difference between the results for each patient in projection images and in radiographs was compared to the distance between the radiocarpal joint and the center of the image (off-centricity) and to deviation of the radius from the Z-axis. Neither showed correlation with differences in measurements between projection images and radiographs.

## Discussion

The correlation between the results in all three modalities was strong. The mean difference and the Bland–Altman 95% confidence intervals for projections images compared to radiographs were broader than for cross sectional images compared to radiographs, but these differences were small and unlikely to have clinical relevance. Interobserver variation was comparable between the difference modalities. Average differences between modalities were comparable to interobserver variation, usually slightly greater.

Radiographs are most commonly used in clinical practice as the basis for radiographic measures. Based on this study, radiological measurements performed on projection images obtained from CBCT scans are comparable to those performed on radiographs and CBCT volumetric images. It is acknowledged that the imaging geometry of the CBCT scanner is different from the radiographic technique, in particular considering the shorter x-ray to detector distance. However, this did not appear to affect the results significantly.

Patient positioning did not affect the accuracy of the measurements in PI's although the angle of the cone beam to the radiocarpal joint changes depending on the distance of the joint from the center of the image. The observed positioning independence may be related to anatomical characteristics of the radiocarpal joint. Owing to the concavity of radial surfaces, PI's could accurately depict the anatomy of the wrist despite the off-centricity. This result of our study might not be applicable to other joints with different morphology.

Our study had some limitations including the modest sample size and heterogeneous patient population (some patients were operatively treated and others conservatively). However, this heterogeneity reflects the normal clinical preference on the used imaging method. Another potential limitation was that, because 37.5% percentage of the fractures had been operated and had normal anatomic features at least partially restored, it may have been easier to perform measurements on them compared to images of untreated fractures.

A potential uncertainty factor could be the differing interpretations owing to different observers choosing different CBCT projection images. However, at least in terms of the most clinically relevant measurements performed in this study, this did not turn out to be a problem. Because the projection images are taken at over 180° around the limb (the detector spins 200° around the imaging target), in some cases observers could potentially choose an image or an opposing one separated by 180°. There were three cases in our study where the two observers chose lateral projections in this way. In one of these cases there was almost no difference in the measurement of volar tilt. Another case represented a severely deformed fracture and the third case was an operated wrist in which the plate partially obscured the joint.

Owing to the lower radiation dose per frame, CBCT projection images exhibit a lower contrast to noise ratio compared to traditional radiographs. Despite this difference, the PI’s could depict anatomical structures sufficiently well for the radiographic measurements assessed here because there are hundreds of images which can be followed to identify structures as a continuum rather than only a few isolated projections. Three-dimensional imaging modalities like CBCT are increasingly used to detect occult fractures and assess fracture morphology, which raises the issue of the necessity of radiographs. It may not be necessary to obtain radiographs of wrist fractures separately for the purpose of radiographic measurements, if a CBCT scan has already been deemed necessary if projections images can be used as a substitute for radiographs. This would reduce the need for additional imaging studies and promote the use of CBCT as a first-line imaging modality. Although typical CBCT scans of the wrist have a higher radiation exposure than plane radiographs the absolute effective dose is very low (in the order of few µSv level) corresponding to a very low radiation risk level [12].

These measurements may however be easier to perform on CBCT projection images as compared to CBCT cross-sectional images because it is not necessary to make reconstructions and evaluations in different planes. A standard cross sectional reformatted image of the radius in the coronal plane might not show the necessary ulnar landmarks for measuring ulnar variance, and additional reformatting of the 3D volume data could be necessary. Three dimensional images are more time-consuming to interpret because it is more difficult to find an optimal reformat plane. Particularly from the clinician’s point-of-view, measurements made on CBCT projection images could be more straightforward especially if clinicians lack time or ready access to the software necessary for additional 3D analysis. Although 3D image analysis improves diagnostic accuracy, both radiologists and clinicians already face an ever-increasing work-load caused by the large amount of data involved in advanced imaging methods. In the future, AI-based methods are likely to automize common measurements directly from the raw-data projection data or from the cross-sectional image data thus facilitating radiologist burden in image analysis [13, 14].

## Conclusions

In conclusion, our study shows that using CBCT projection images for wrist imaging is pragmatic and offers sufficient accuracy for radial measurements reducing the need for radiographs and for analysis of three-dimensional images for the purpose of making common radiographic measurements.

### Supplementary Information

**Additional file 1. **The supplementary file "DATA_measurements.xlsx" is an Excel file of the data analyzed in this study, that is the measurements made by the two reviewers of the images.

## Data Availability

All data generated or analyzed during this study are included in this published article (and its supplementary information files).

## Abbreviations

3D: Three dimensional
AI: Artificial intelligence
CBCT: Cone beam computed tomography
CSI: Cross sectional images
CT: Computed tomography
PI: Projection images
RI: Radial inclination
UV: Ulnar variance
VT: Volar tilt
